# Salidroside Ameliorates Polycystic Ovary Syndrome in Mice by Regulating the AKT/NF‐κB/NLRP3‐HAS2 Axis

**DOI:** 10.1002/fsn3.71692

**Published:** 2026-04-04

**Authors:** Jing‐hang Li, Shu‐Ming Shi, Li‐Ying Liu, Yu‐Yan He, Guan‐Lin Jia, Lin‐Yi Qu, Zhi‐Chao Chi, Jia‐hui Leng, Hong‐jie Xu, Tian Lan, Dong‐lei Yan, Il‐keun Kong, Xian‐Feng Yu, Ming‐Jun Zhang, Yong‐Xun Jin

**Affiliations:** ^1^ Jilin Provincial Key Laboratory of Animal Model, College of Animal Science Jilin University Changchun China; ^2^ Animal Genome Editing Technology Innovation Center, Jilin Province, College of Animal Science Jilin University Changchun China; ^3^ Division of Applied Life Science (BK21 Four), Department of Animal Science Gyeongsang National University Jinju Republic of Korea

**Keywords:** AKT, HAS2, inflammation, PCOS, salidroside

## Abstract

Salidroside, a bioactive compound from *Rhodiola rosea* , shows potential in managing polycystic ovary syndrome (PCOS), a common endocrine‐metabolic disorder. This study explored its therapeutic effects and mechanisms using a dehydroepiandrosterone (DHEA)‐induced PCOS mouse model and granulosa cells (GCs). Salidroside was found to restore estrous cyclicity, improve ovarian morphology, and rebalance serum hormone levels. Mechanistically, it suppressed ROS‐mediated AKT/NF‐κB signaling, inhibited NLRP3 inflammasome activation, and reduced proinflammatory cytokines. Network pharmacology and molecular docking identified AKT as a core target, validated by CETSA and DARTS assays. Furthermore, salidroside disrupted NLRP3‐driven latent TGF‐β1 (LAP‐TGFβ1) activation, downregulated TGF‐β‐SMAD2/3 signaling, and attenuated ovarian fibrosis along with abnormal hyaluronan synthase 2 (HAS2) expression. These results highlight salidroside as a promising natural candidate for alleviating PCOS through coordinated anti‐inflammatory and antifibrotic mechanisms.

## Graphical abstract

Salidroside ameliorates PCOS via inhibiting the ROS‐AKT/NF‐κB axis and suppressing NLRP3 inflammasome activation. It disrupts the NLRP3–LAP‐TGFβ1 interaction to block TGF‐β‐SMAD2/3 signaling and consequent ovarian fibrosis. Together with hyaluronan synthase 2 (HAS2) downregulation, these effects alleviate inflammation and hyaluronan overaccumulation.

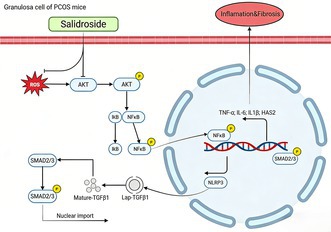

## 1 Introduction

Polycystic ovary syndrome (PCOS) is one of the most prevalent endocrine‐metabolic disorders among women of reproductive age. It is clinically characterized by hyperandrogenism, ovulatory dysfunction, and polycystic ovarian morphology and is frequently accompanied by obesity, insulin resistance, and infertility (Norman et al. 2007; Berni et al. 2021). Notably, PCOS patients exhibit significantly elevated risks of long‐term complications, including endometrial carcinoma and cardiovascular diseases, which profoundly compromise their health and quality of life (Berni et al. 2021; Li et al. 2025).

Emerging evidence has revealed a unique pathological microenvironment in PCOS ovaries, where chronic low‐grade inflammation and oxidative stress form a vicious cycle. This cycle disrupts extracellular matrix (ECM) homeostasis, leading to aberrant hyaluronan (HA) deposition (He et al. 2024). Current therapeutic strategies are tailored to symptomatic management and provide only transient clinical relief; they lack sustained efficacy and may even exacerbate metabolic disturbances (Norman et al. 2007), underscoring the urgent need for novel PCOS treatment paradigms.

Salidroside, a naturally occurring phenolic glycoside, possesses multiple pharmacological properties including anti‐inflammatory, antioxidant, and proangiogenic effects (Han et al. 2022). It has been demonstrated to effectively treat cardiovascular diseases and enhance adaptation to high‐altitude hypoxia (Han et al. 2022; Jiang et al. 2022). Our previous studies revealed that salidroside promotes in vitro embryonic development of porcine oocytes by reducing oxidative stress (Shi et al. 2023) and also improves ovarian vascular remodeling in aged mice (Mu et al. 2025). Furthermore, salidroside ameliorates glucose metabolism by increasing insulin sensitivity and reducing lipid accumulation through the promotion of fatty acid oxidation, thereby alleviating obesity and insulin resistance in diabetic mice (Zhu et al. 2023). Salidroside also inhibits the nuclear translocation of NF‐κB p65, reversing the pathological loss of retinal ganglion cells in diabetic rats (Feng et al. 2024). Collectively, these findings suggest the therapeutic potential of salidroside for the treatment of PCOS. The benefits of salidroside in metabolic and reproductive contexts have been partially validated. However, its precise efficacy and underlying mechanisms require further investigation.

The present study aimed to elucidate the multitarget mechanisms by which salidroside ameliorates PCOS pathogenesis. Our experimental data demonstrated that salidroside suppresses the ROS‐mediated activation of the AKT/NF‐κB signaling cascade, thereby inhibiting NLRP3 inflammasome assembly and reducing proinflammatory cytokine expression. A key novel finding is that salidroside impedes pathological interactions between NLRP3 and latent transforming growth factor‐β1 (LAP‐TGF‐β1) precursors, effectively inhibiting fibrogenic signaling activation. Molecular profiling revealed that salidroside mediated hyaluronan synthase 2 (HAS2) downregulation, which normalized HA overaccumulation within inflammatory microenvironments, ultimately alleviating granulosa cell oxidative damage and reversing fibrotic phenotypes. These discoveries systematically delineated the dual anti‐inflammatory and antifibrotic actions of salidroside in preserving ovarian function, providing a mechanistic foundation for the development of innovative PCOS therapeutics.

## 2 Materials and Methods

### 2.1 Animal Experiments

A total of 50 female C57BL/6 mice (3 weeks old) were housed under SPF conditions at a controlled temperature (22°C ± 1°C) and relative humidity (60% ± 5%) on a 12‐h light/dark cycle. Following 7 days of acclimatization, the animals were randomly allocated to the following five experimental groups (*n* = 10): Control (CON) group, dehydroepiandrosterone (DHEA)‐induced group, and three treatment groups receiving different doses of salidroside (10, 20, and 40 mg/kg).

**FIGURE 1:**
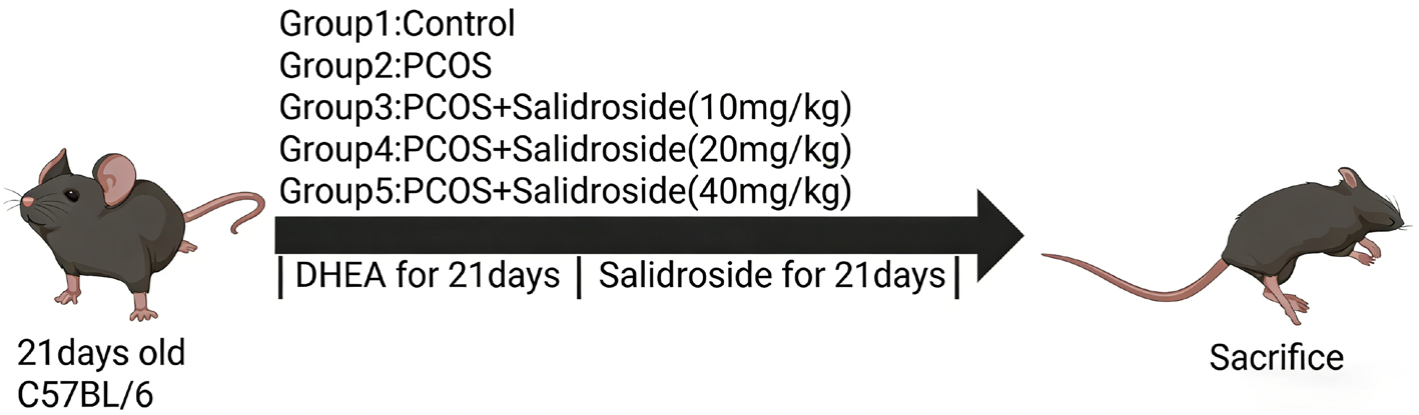
Animal experimental protocol.

The model was established by daily subcutaneous injection of DHEA (60 mg/kg in vehicle (olive oil), 0.1 mL/10 g, LKT), whereas the CON group received an equivalent volume of olive oil alone. The treatment schedule for the mice is shown in Figure 1. After 21 days of model establishment, the therapeutic interventions were initiated. Although the CON and DHEA groups were administered normal saline (0.2 mL/10 g) via oral gavage, the treatment groups received corresponding doses of salidroside (Yuanye) dissolved in saline daily for 21 consecutive days. Body weight and behavioral parameters were monitored throughout the study. All experimental protocols were approved by the Ethical Welfare Committee of Jilin University (no. SY202409305) and were conducted in accordance with the ARRIVE 2.0 guidelines.

### 2.2 Assessment of the Estrous Cycle

During the final 10 days of the experiment, the vaginal secretions from each mouse were collected, fixed in methanol, air‐dried, and subsequently stained with Wright–Giemsa solution (Beyotime). The predominant cell types observed in the smears were analyzed under a microscope to determine the estrous cycle stage.

### 2.3 Hematoxylin and Eosin (H&E) and Masson's Staining

Ovarian tissues were fixed in 4% paraformaldehyde for 24 h, dehydrated through a graded ethanol series, cleared in xylene, and embedded in paraffin wax. Serial sections (6 μm thick) were obtained using a rotary microtome and heat‐immobilized at 60°C. After staining with H&E and Masson's trichrome, the sections were coverslipped with synthetic mounting medium for bright‐field microscopic analysis of the histomorphological features.

### 2.4 Isolation of GCs

Three weeks old female C57BL/6 mice were intraperitoneally administered 10 IU of pregnant mare serum gonadotropin (PMSG), and the ovaries were aseptically collected 48 h later. GCs were released by mechanical disruption of ovarian follicles using a 1 mL syringe needle. The suspended cells were seeded into 6‐well plates containing complete DMEM/F12 medium (supplemented with 10% fetal bovine serum and 1% penicillin–streptomycin) and cultured in a humidified incubator at 37°C with 5% CO_2_. The medium was changed every 48 h.

### 2.5 GC Treatment

GCs were treated with salidroside (100 μM; Sigma) for 12 h. The cotreatments included the AKT agonist SC79 (10 μM; Beyotime), the reactive oxygen species (ROS) inhibitor N‐acetylcysteine (NAC; 20 μM; MCE), the AKT inhibitor MK‐2206 (10 μM; MCE), the NF‐κB activator phorbol 12‐myristate 13‐acetate (PMA; 5 ng/mL; MCE), and the TGF‐β agonist SRI‐011381 (10 μM; MCE). All the reagents were dissolved in dimethyl sulfoxide (DMSO) and diluted to a final DMSO concentration < 0.1%.

To overexpress NLRP3 in GCs, GCs were transfected with the pcDNA3.1‐NLRP3 plasmid using Lipofectamine 3000. Subsequent treatments were performed 48 h posttransfection. DMSO vehicle controls and untreated blank controls were included in all the experiments.

### 2.6 Enzyme‐Linked Immunosorbent Assay (ELISA)

Serum samples collected via retro‐orbital bleeding were centrifuged at 3000 × *g* and 4°C for 15 min. The concentrations of testosterone (T), estrogen (E2), progesterone (P4), follicle‐stimulating hormone (FSH), luteinizing hormone (LH), and inflammatory cytokines, including interleukin‐6 (IL‐6), interleukin‐1 beta (IL‐1β), and tumor necrosis factor alpha (TNF‐α), were determined using ELISA kits (Elabscience).

### 2.7 Western Blot Analysis

Protein extracted from the ovaries and GCs was quantified by a BCA (Abbkine) assay, separated via sodium dodecyl sulfate–polyacrylamide gel electrophoresis (SDS–PAGE), and transferred onto PVDF membranes. The membranes were blocked with 5% skim milk for 1 h at room temperature, followed by incubation with primary antibodies (listed in Table S1) at 4°C overnight. After the membranes were washed with TBST, an HRP‐conjugated goat anti‐rabbit IgG secondary antibody (1:5000; Epizyme) was applied for 2 h at room temperature. The signals were detected via an ECL hypersensitive substrate.

### 2.8 RT–qPCR

Total RNA was extracted from ovaries and GCs using TRIzol reagent. cDNA was synthesized by reverse transcription, followed by quantitative PCR amplification using SYBR Green Premix (Servicebio). Gene expression levels were analyzed via the $2^{-∆∆C_{t}}$ method. The specific sequences of primers used are listed in Table S2.

### 2.9 Network Pharmacology Analysis

The potential binding targets of salidroside were predicted through integrative mining of multiple databases including the Comparative Toxicogenomics Database (CTD; http://ctdbase.org/), HERB (http://herb.ac.cn/), the Encyclopedia of Traditional Chinese Medicine (ETCM; http://www.tcmip.cn/ETCM/), and PharmMapper (http://www.lilab‐ecust.cn/pharmmapper/). Predictions from each database were initially filtered based on their respective built‐in confidence metrics or ranking systems to retain high‐confidence candidates. Consensus targets were then identified by intersecting the predictions from each database. This consensus strategy enhances specificity and mitigates false positives by requiring targets to be supported by multiple independent sources. PCOS‐related genes were retrieved from GeneCards (https://www.genecards.org) and Online Mendelian Inheritance in Man (OMIM; https://omim.org/) using “polycystic ovary syndrome” and “ *Homo sapiens* ” as the search terms.

Intersection analysis of the salidroside targets and PCOS‐associated genes was performed using Venny2.1 (https://bioinfogp.cnb.csic.es/tools/venny/) to generate Venn diagrams. Overlapping targets were uploaded to the STRING platform (https://string‐db.org/) for protein–protein interaction (PPI) network construction, with the organism restricted to *H. sapiens* and an interaction confidence score threshold > 0.9. Functional enrichment analysis was conducted via Metascape (https://metascape.org/), which covers Gene Ontology (GO) terms and Kyoto Encyclopedia of Genes and Genomes (KEGG) pathways. The enrichment results were visualized using the Weishengxin online platform (http://www.bioinformatics.com.cn/).

### 2.10 Molecular Docking

The crystal structure of the human protein serine/threonine‐protein kinase 1 (AKT1) (PDB ID: 3O96) was retrieved from the RCSB Protein Data Bank (RCSB PDB; https://www.rcsb.org/) with screening criteria of X‐ray crystallography (resolution 2.8 Å) and experimentally verified active conformation. The structure was processed using PyMOL 2.5.2 to remove crystallographic water molecules and cocrystallized ligands while retaining key amino acid residues in the active pocket. Molecular docking was performed with AutoDockTools 1.5.7 and AutoDock Vina 4.2. For protein–protein docking, the HDOCK online server (http://hdock.phys.hust.edu.cn/) was utilized to dock NLRP3 (PDB ID: 8WSM) and latent transforming growth factor beta 1 (LAP‐TGFβ1, PDB ID: 8VSC). The possible binding modes were explored using the fast Fourier transform (FFT) algorithm and evaluated by a knowledge‐based scoring function. The system provided the top 10 docking poses for visualization and the top 100 poses for download. The complex with the lowest (most negative) docking score was selected as the final model. Visualization was performed using PyMOL and Discovery Studio software.

### 2.11 Immunofluorescence

GCs were fixed with 4% paraformaldehyde for 15 min at room temperature, permeabilized with 0.1% Triton X‐100/PBS for 10 min, and blocked with 3% bovine serum albumin (BSA) for 30 min. The GCs were incubated overnight at 4°C with the following primary antibodies: rabbit anti‐p65 (1:200 dilution) for NF‐κB p65 nuclear translocation analysis or a mixture of rabbit anti‐NLRP3 and mouse anti‐Flag antibodies for colocalization studies. After the cells were washed with PBS, they were incubated with species‐specific Alexa Fluor‐conjugated secondary antibodies at 37°C for 1–2 h in the dark. Nuclei were stained with DAPI for 5 min, and slides were mounted for fluorescence microscopy imaging. NF‐κB p65 nuclear translocation was quantified by calculating the nuclear‐to‐cytoplasmic fluorescence intensity ratio. For NLRP3/Latent‐TGF‐β1 colocalization analysis, the NLRP3 and Flag‐Latent‐TGF‐β1 signals were visualized in the green and red channels, respectively.

### 2.12 ROS Assay

Cellular total ROS levels were quantified using a commercial ROS detection kit (Beyotime). Following trypsinization, the cells were processed in accordance with the manufacturer's protocol. Briefly, the fluorescent probe DCFH‐DA (10 mM stock) was diluted 1:1000 in serum‐depleted medium, and the cell pellets were resuspended in the probe‐containing medium followed by incubation at 37°C for 20 min. The cells were washed three times with serum‐free medium to remove residual unbound probes. Nonfluorescent DCFH undergoes ROS‐dependent oxidation to yield fluorescent 2′,7′‐dichlorofluorescein (DCF). Quantification of the fluorescence intensity was conducted via flow cytometry.

### 2.13 Measurement of Antioxidant Enzyme Activity

The activities of superoxide dismutase (SOD) and catalase (CAT) in the ovarian tissue homogenates and GCs lysates were determined using commercial kits (Beyotime). For SOD detection, the supernatant obtained after centrifugation was mixed with WST‐8/enzyme working solution, and the absorbance of the mixture was measured at 450 nm following the addition of the reaction starter. For the analysis of CAT activity, the supernatant was treated with detection buffer containing hydrogen peroxide (H_2_O_2_) and the chromogenic substrate, after which the change in absorbance was measured at 520 nm.

### 2.14 GSH Assay

The content of reduced glutathione (GSH) and the GSH/oxidized glutathione (GSSG) ratio in the ovarian tissue homogenates and GC lysates were quantified using a commercial GSH/GSSG assay kit (Beyotime). In accordance with the manufacturer's protocol, supernatants obtained by centrifugation were incubated with reaction buffer containing glutathione reductase (GR) and 5,5′‐dithiobis (2‐nitrobenzoic acid) (DTNB) at 25°C for 5 min. Subsequently, NADPH solution was added to the reaction system under scavenger‐supplemented or scavenger‐depleted conditions (to differentiate between GSH and GSSG), and kinetic measurements of the absorbance at 412 nm were performed at 5 min intervals using a microplate reader. The final calculated GSH concentrations and GSH/GSSG ratios were derived from the standard curve established using reference standards and the algorithmic formulae specified in the technical manual.

### 2.15 Malondialdehyde (MDA) Assay

MDA levels were quantified using a lipid peroxidation assay kit (Beyotime) following the manufacturer's protocol. Briefly, tissue homogenates or cell lysates were processed alongside reference standards, and the absorbance was measured at 532 nm using a microplate reader. The MDA concentrations, initially calculated in nmol/mL, were normalized to the total protein content (μmol/mg protein) after BCA assay‐based protein quantification.

### 2.16 Co‐Immunoprecipitation (Co‐IP)

PPIs were analyzed via a coimmunoprecipitation assay kit (Servicebio) following the manufacturer's standardized protocol. For immunoprecipitation, anti‐Latent‐TGFβ1 and anti‐NLRP3 antibodies were used as specific capture reagents, with species‐matched IgG serving as an isotype control. Subsequent immunoblot analysis was performed with the same primary antibodies against Latent‐TGFβ1 and NLRP3 for specific antigen detection. All procedures adhered strictly to the manufacturer's recommended guidelines.

### 2.17 Cellular Thermal Shift Assay (CETSA)

GCs were lysed, divided into two equal aliquots and flash‐frozen in liquid nitrogen. Following 2 h of incubation with either salidroside or DMSO, the lysates were further partitioned into 10 aliquots and subjected to gradient heat treatment at temperatures ranging from 42°C to 62°C for 3 min each. The heated samples were subsequently centrifuged, and the resulting supernatants were collected for Western blot analysis.

### 2.18 Drug Affinity Responsive Target Stability (DARTS) Assay

The total protein extracts from the GCs were diluted with TNC buffer (50 mM Tris–HCl [pH 8.0], 50 mM NaCl, and 10 mM CaCl_2_) followed by treatment with either salidroside (Sal) or DMSO. After 1 h of incubation at room temperature, pronase (25 μg/mL; MeilunBio) was added, and the mixture was maintained at 37°C for 30 min. The reaction was terminated by the addition of SDS–PAGE loading buffer, and protein detection was subsequently performed via Western blotting.

### 2.19 Statistical Analysis

The flow cytometry data were analyzed using FlowJo software (version X.10.0.7r2). Statistical analyses were performed and plots were constructed with GraphPad Prism 9.5, and the data are expressed as the means ± SDs. Differences among multiple groups were compared by one‐way analysis of variance (ANOVA), and *p* < 0.05 was considered to indicate statistical significance. All experiments were independently repeated at least three times.

## 3 Results

### 3.1 Salidroside Alleviates Symptomatic Manifestations in PCOS MICE

**FIGURE 2:**
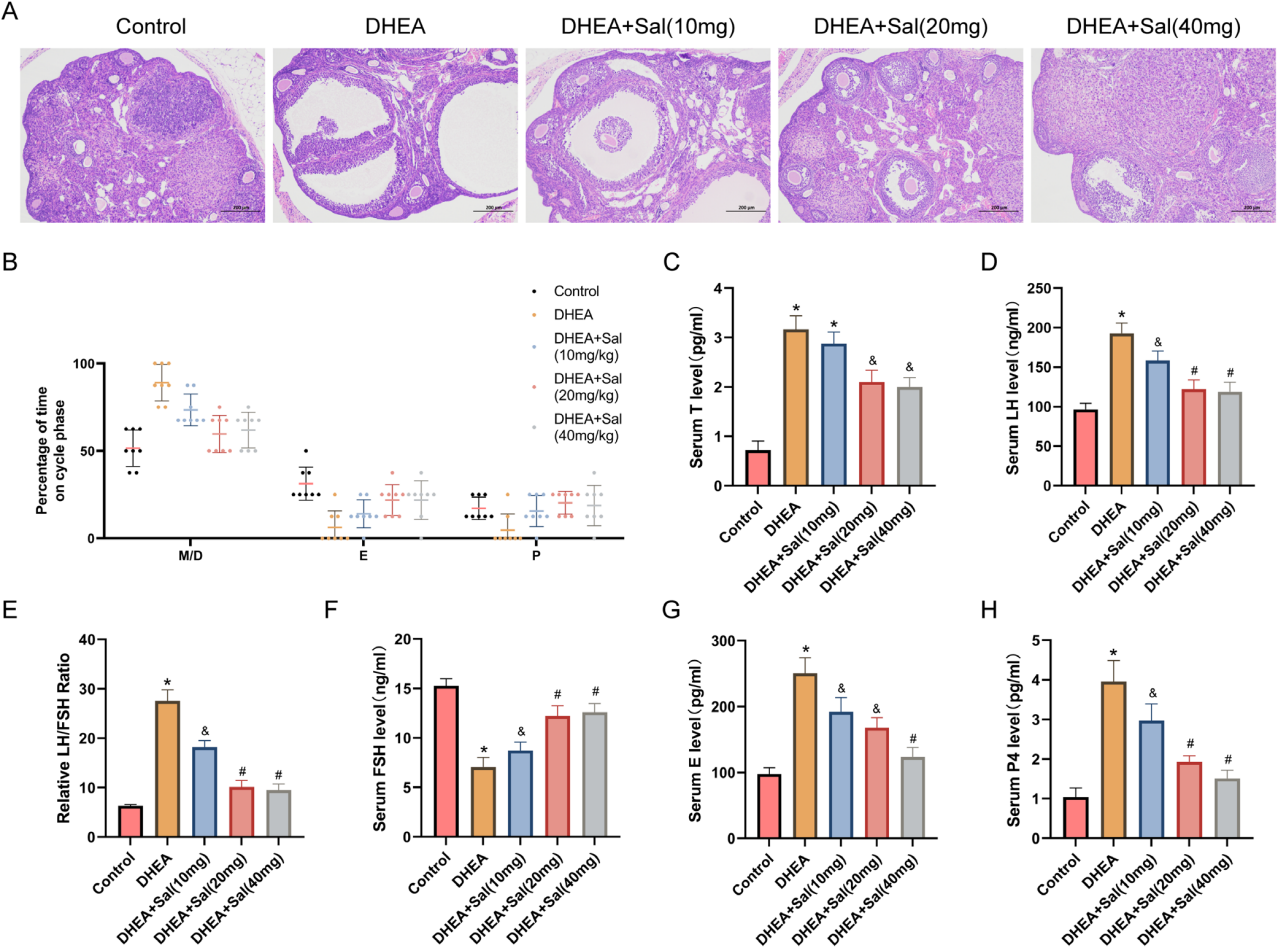
Salidroside alleviates PCOS symptoms in mice. (A) H&E staining revealed that salidroside improved ovarian morphology in PCOS mice (scale bar = 200 μm). (B) Percent duration of each estrous cycle phase in PCOS mice after 21 consecutive days of salidroside treatment. E, estrus; M/D, metestrus/diestrus; P, proestrus. (C–F) Effects of salidroside on serum T, LH, and FSH levels and the LH/FSH ratio (*n* = 10 per group). (G, H) Effects of salidroside on serum E2 and P4 levels (*n* = 10 per group). Values represent means ± SD. *p < 0.05 vs. the control group; ^&^ *p* < 0.05 vs. the DHEA‐treated group; and ^#^ *p* < 0.05 vs. the Sal (10 or 20 mg/kg) + DHEA groups.

Salidroside administration dose‐dependently ameliorated estrous cycle disruption in PCOS model mice (Figure 2A). The 10 mg/kg dose partially restored cyclicity, whereas the 20 and 40 mg/kg doses effectively normalized the estrous rhythm. Moreover, histopathological analysis revealed characteristic PCOS features in the model group ovaries including increased cystic/atretic follicles and a reduced corpus luteum count (H&E staining, Figure 2B). Salidroside treatment progressively reversed these abnormalities. In particular, 10 mg/kg salidroside partially improved follicular development, whereas 20 and 40 mg/kg salidroside restored ovarian morphology to near‐normal levels, with a decreased cystic/atretic follicle ratio and restored corpus luteum numbers. Moreover, hormonal profiling demonstrated that salidroside significantly attenuated endocrine dysregulation (Figure 2C–F). The increase in serum T levels in PCOS mice was dose‐dependently reduced by salidroside, with 40 mg/kg salidroside restoring T to within the normal range. Salidroside treatment also restored LH levels, decreased the LH/FSH ratio, and restored FSH levels to control levels. Furthermore, the excessive secretion of E2 and P4 in PCOS mice was effectively suppressed by salidroside administration (Figure 2G,H).

### 3.2 Salidroside Alleviates Antioxidant‐Dependent Inflammation

**FIGURE 3:**
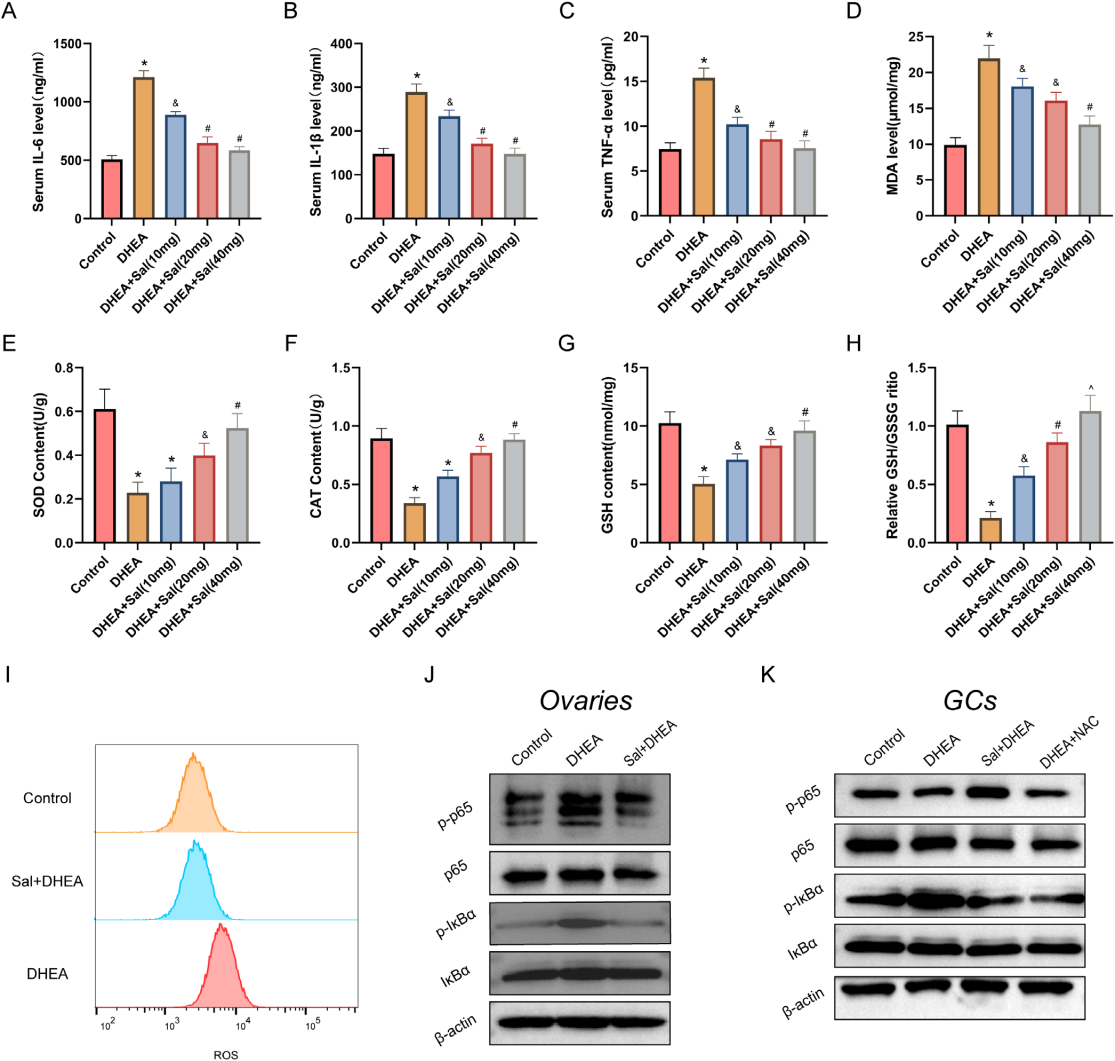
Effects of salidroside on inflammation and oxidative stress in PCOS mice. (A–C) Effects of salidroside on the serum IL‐6, IL‐1β, and TNF‐α levels in PCOS mice (*n* = 10 per group). (D) Ovarian MDA contents (*n* = 3 per group). (E, F) Ovarian SOD and CAT activities (*n* = 3 per group). (G, H) Ovarian GSH content and GSH/GSSG ratio (*n* = 3 per group). (I) ROS levels in GCs. (J, K) Western blot analysis of phospho‐IκBα, total IκBα, phospho‐p65, and p65 protein levels in the ovaries of PCOS mice and in GCs. Values represent means ± SD. *p < 0.05 vs. the control group; ^&^ *p* < 0.05 vs. the DHEA‐treated group or the DHEA + Sal (10 mg/kg) group; ^#^ *p* < 0.05 vs. the Sal (10 or 20 mg/kg) + DHEA group; and ^*p* < 0.05 vs. the DHEA + Sal (20 mg/kg) group.

Chronic inflammation has been confirmed as a critical factor in the pathogenesis of PCOS (Jin et al. 2024). Salidroside treatment significantly reduced the serum levels of the proinflammatory cytokines IL‐6, IL‐1β, and TNF‐α in PCOS mice (Figure 3A–C). Analysis of ovarian tissue homogenates revealed a marked increase in the MDA content in PCOS mice, which was effectively reversed by salidroside intervention (Figure 3D). Furthermore, salidroside ameliorated redox imbalance by enhancing the antioxidant defense system, as evidenced by the significantly increased activities of SOD and CAT, along with the elevated GSH content and GSH/GSSG ratio (Figure 3E–H). Analogous improvements in antioxidant capacity were observed in GCs (Figure S1), accompanied by a significant reduction in intracellular ROS levels following salidroside treatment (Figure 3I). In addition, treatment with 40 mg/kg salidroside had a dose‐dependent effect on alleviating both inflammation and oxidative stress. Therefore, this dosage was selected for subsequent mechanistic investigations.

Previous studies have suggested that the anti‐inflammatory effects of salidroside are closely associated with NF‐κB signaling pathway suppression (Jiang et al. 2022; Feng et al. 2024; Lei et al. 2024). Western blot analysis confirmed that salidroside effectively inhibited the aberrant phosphorylation of IκBα and NF‐κB subunit p65 in the ovarian tissues of PCOS mice (Figure 3J). In GCs, the addition of the ROS inhibitor NAC resulted in decreased phosphorylation patterns similar to those observed in the salidroside‐treated groups (Figure 3K), further supporting the regulatory role of the ROS/NF‐κB axis (Zhao et al. 2024). Collectively, these data suggest that salidroside likely exerts therapeutic effects by inhibiting DHEA‐induced oxidative stress and NF‐κB phosphorylation.

### 3.3 Network Pharmacology Identification of AKT as a Key Target of Salidroside in PCOS Treatment

**FIGURE 4:**
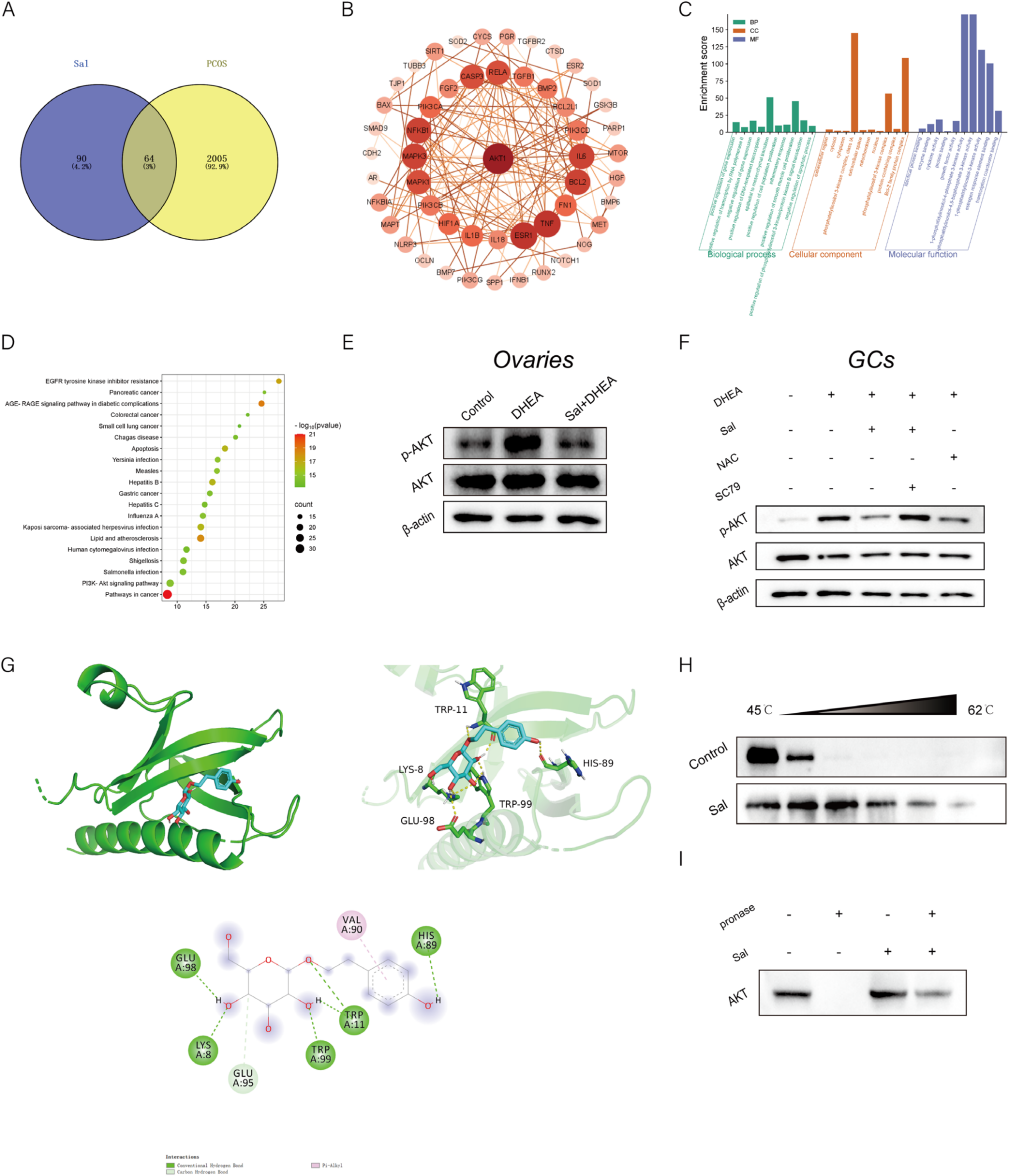
Bioinformatics mining and validation of core genes shared by PCOS and salidroside. (A) Venn diagram of the PCOS‐Sal intersecting targets. (B) PPI network of the core targets. (C) GO analysis of the PCOS‐Sal intersecting targets. (D) KEGG pathway enrichment analysis of PCOS‐Sal intersecting targets. (E, F) Western blot analysis of phospho‐AKT and total AKT protein levels in the ovaries of PCOS mice and in GCs. (G) Molecular docking of Sal and AKT1. (H, I) DARTS assay and CETSA for AKT in GCs.

To systematically investigate the therapeutic mechanism of Sal in PCOS, the present study identified 154 candidate targets of Sal through integrative mining of the CTD, HERB, ETCM, and PharmMapper databases. Moreover, 2069 PCOS‐related therapeutic targets were retrieved from the GeneCards and OMIM databases. Venn diagram analysis revealed 64 overlapping targets (Figure 4A), suggesting their potential involvement in mediating the regulatory effects of Sal on PCOS.

Topological analysis of the PPI network highlighted core targets (Figure 4B) including B‐cell lymphoma 2 (BCL2), AKT1, nuclear factor kappa B subunit 1 (NFκB1), mitogen‐activated protein kinase 3 (MAPK3), interleukin 6 (IL6), estrogen receptor 1 (ESR1), and tumor necrosis factor (TNF), all of which presented high degree values. These findings indicated their pivotal roles in the anti‐PCOS activity of Sal. KEGG pathway enrichment analysis demonstrated significant enrichment of overlapping targets in PCOS‐associated signaling pathways, particularly the PI3K/Akt signaling pathway (Figure 4D). Notably, AKT1 presented the highest degree value in the network analysis. Given that Sal effectively suppresses aberrant activation of the NFκB signaling pathway in PCOS models and that AKT acts as an upstream regulator of NFκB with established proinflammatory roles across various pathological conditions (Kong et al. 2025; Lin et al. 2023), we hypothesized that Sal may ameliorate PCOS progression through targeted modulation of the Akt/NFκB signaling axis, thereby interrupting inflammatory cascades.

To validate this hypothesis, Western blot analysis was performed and revealed a marked increase in phosphorylated AKT (p‐AKT) levels in PCOS ovaries, which was effectively reversed by Sal treatment (Figure 4E). Consistent results were observed in GCs (Figure 4F). Notably, the aberrant phosphorylation of AKT in DHEA‐induced GCs was also significantly suppressed upon cotreatment with the ROS inhibitor NAC. To investigate the potential interaction between Sal and AKT1, molecular docking simulations were performed using AutoDock Vina. The analysis predicted a potential binding mode of Sal within the hydrophilic pocket of AKT1, with a calculated binding energy of −7.6 kcal/mol (Figure 4G; values < −5 kcal/mol generally indicate strong binding). Furthermore, a CETSA and DARTS experiment confirmed that Sal treatment significantly enhanced the structural stability of the AKT1 protein (Figure 4H,I), providing biophysical evidence for their specific interaction.

### 3.4 Salidroside Attenuates Inflammatory Responses via Modulation of the AKT/NF‐κB Signaling Pathway

**FIGURE 5:**
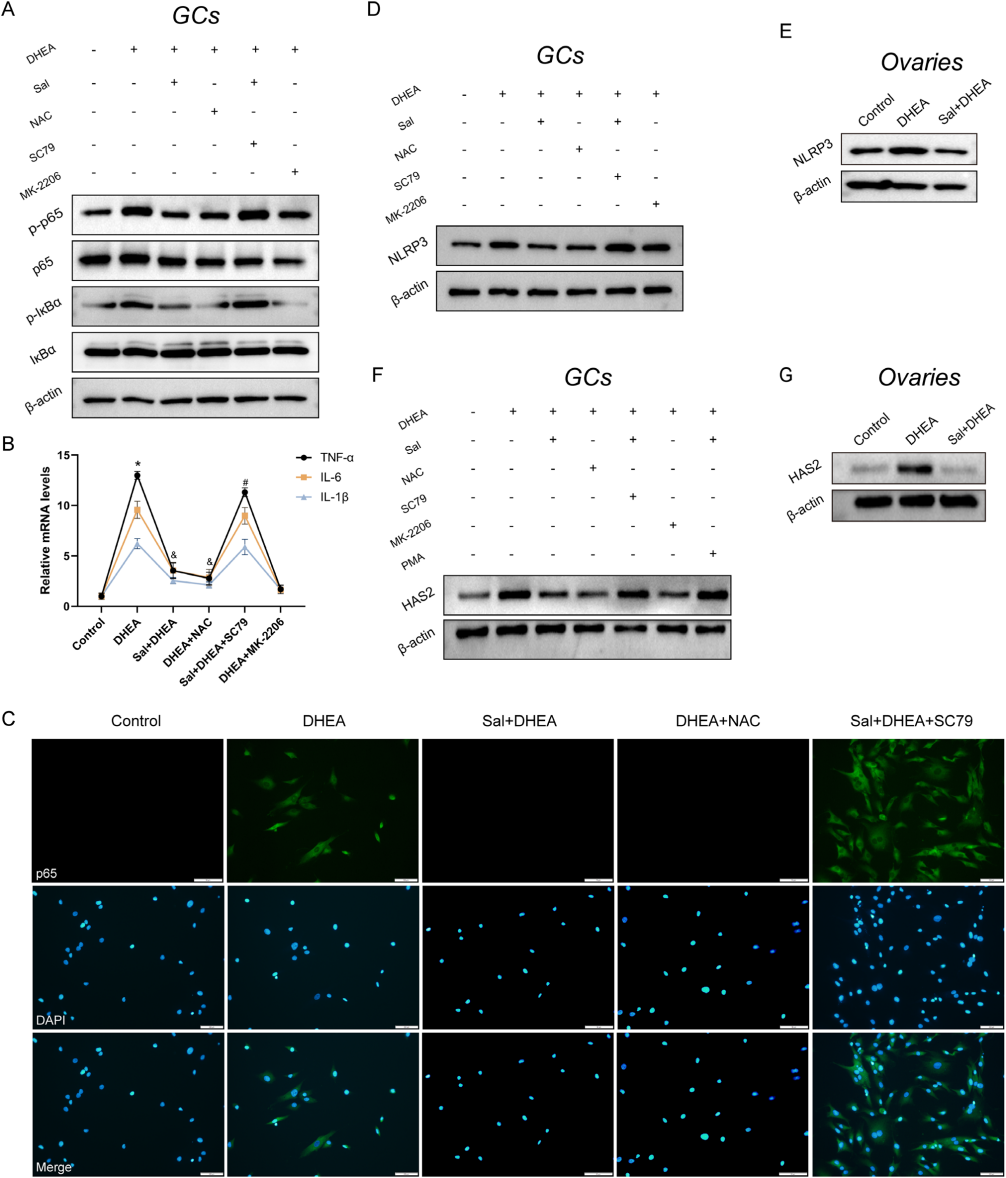
Salidroside alleviates inflammation in PCOS by modulating AKT signaling. (A) Western blot analysis of phospho‐p65, total p65, phospho‐IκBα, and total IκBα in GCs. (B) mRNA levels of IL‐1β, IL‐6, and TNF‐α in GCs (*n* = 3 per group). (C) Immunofluorescence analysis of p65 nuclear translocation in GCs. (D, E) Western blot analysis of NLRP3 protein expression in GCs and in the ovaries of PCOS mice. (F, G) Western blot analysis of HAS2 protein levels in GCs and in the ovaries of PCOS mice. Values represent means ± SD. *p < 0.05 vs. the control group; ^&^ *p* < 0.05 vs. the DHEA‐treated group; and ^#^ *p* < 0.05 vs. the DHEA + NAC‐treated group.

To investigate whether salidroside exerts anti‐inflammatory effects via the AKT/NF‐κB signaling axis, we systematically examined the functional role of AKT in GCs. Western blot analysis demonstrated that the AKT inhibitor MK‐2206 significantly suppressed DHEA‐induced phosphorylation of factors in the NF‐κB pathway, and cotreatment with the AKT agonist SC79 reversed the inhibitory effects of salidroside, indicating that AKT plays a pivotal regulatory role in this mechanism (Figure 5A). In DHEA‐induced GCs, both the ROS inhibitor NAC and salidroside significantly decreased the mRNA expression of proinflammatory cytokines including IL‐6, IL‐1β, and TNF‐α (Figure 5B).

Immunofluorescence analysis revealed that DHEA induction significantly increased p65 nuclear translocation efficiency, which was strongly inhibited by Sal intervention. Cotreatment with SC79 partially restored p65 nuclear localization, further confirming that AKT activation counteracts the regulatory effects of Sal (Figure 5C) and suggesting that AKT signaling may contribute to the anti‐inflammatory effects of Sal.

Mechanistic investigations revealed the crucial involvement of the NLRP3 inflammasome as a downstream mediator of the NF‐κB pathway in the inflammatory processes associated with PCOS (Feng et al. 2024; Xiang et al. 2023; Liu et al. 2024). The present findings revealed that there was a significant increase in NLRP3 expression in both DHEA‐induced GCs and mouse ovaries. Remarkably, salidroside effectively suppressed the excessive activation of NLRP3 in GCs, and the regulation of NLRP3 was notably influenced by both the activation and suppression of AKT signaling pathways (Figure 5D,E).

Emerging evidence suggests that HAS2 is a modulator of ECM remodeling in response to inflammation (He et al. 2024; Rowley et al. 2020). Western blot analysis revealed the pathological upregulation of HAS2 in the PCOS model, which was significantly reversed following treatment with salidroside. Moreover, the NF‐κB agonist PMA counteracted the therapeutic effects of salidroside on abnormal HAS2 expression (Figure 5G,H), indicating that HAS2 may play a pivotal role as a central molecule at the intersection of AKT/NF‐κB signaling. In summary, these findings indicated that salidroside may systematically regulate the inflammatory microenvironment in PCOS by specifically targeting the AKT/NF‐κB/NLRP3 signaling network.

### 3.5 NLRP3‐Driven TGF‐β‐SMAD2/3 Activation Mediates the Antifibrotic Effects of Salidroside in PCOS Ovaries

**FIGURE 6:**
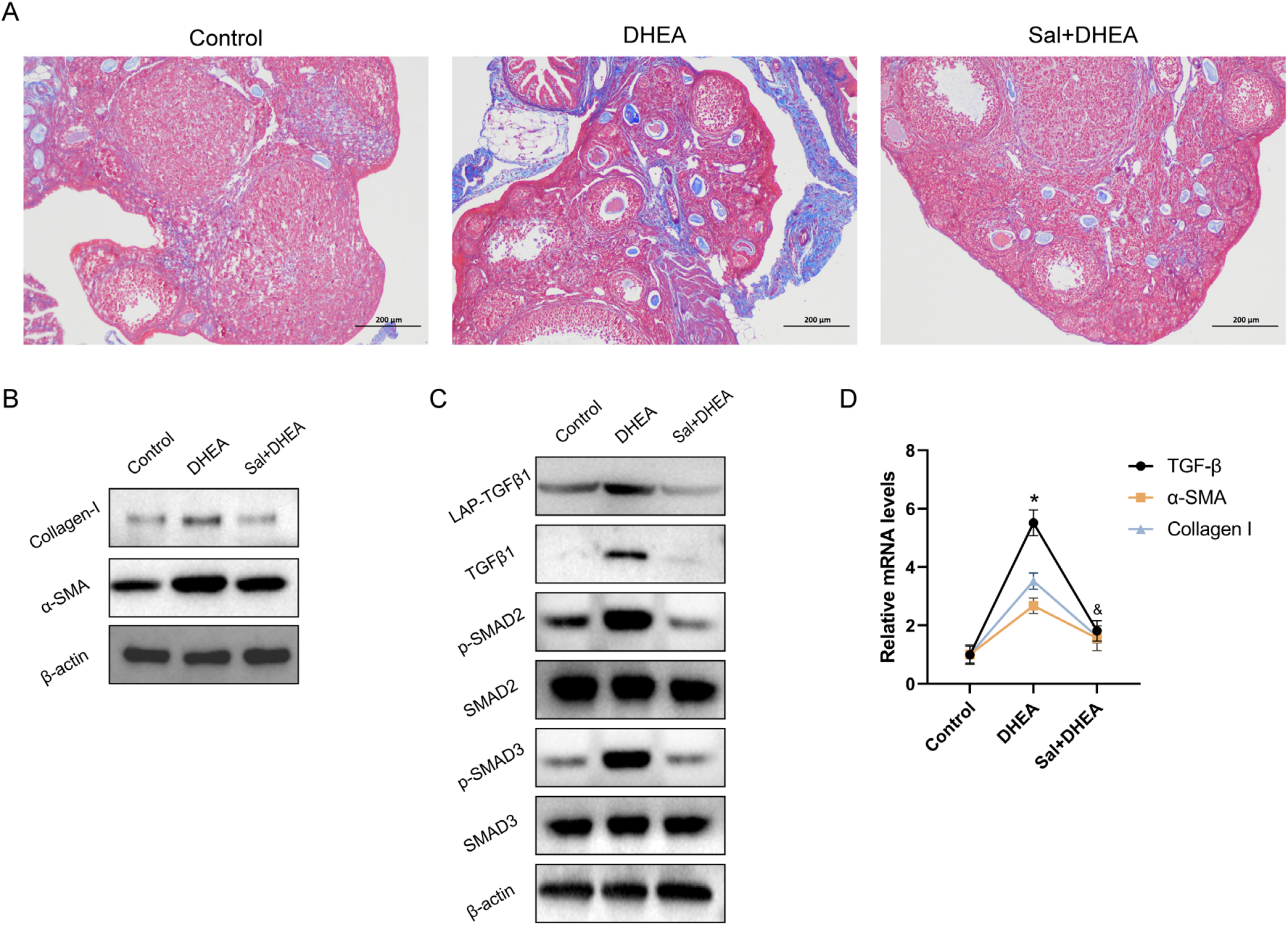
Salidroside alleviates ovarian fibrosis in PCOS mice. (A) Masson staining indicating fibrosis in mouse ovaries (scale bar = 200 μm). (B) Western blot analysis of Collagen I and α‐SMA protein levels in the ovaries of PCOS mice. (C) Western blot analysis of the protein levels of TGF‐β1, LAP‐TGF‐β1, phospho‐SMAD2, total SMAD2, phospho‐SMAD3, and total SMAD3 in the ovaries of PCOS mice. (D) mRNA levels of Collagen I, α‐SMA, and TGF‐β in the ovaries of PCOS mice (*n* = 3 per group). Values represent means ± SD. *p < 0.05 vs. the DHEA group.

Activation of the NLRP3 inflammasome in ovarian tissue has been implicated in both chronic inflammation and fibrotic progression (Xiang et al. 2023; Liu et al. 2024). Masson's trichrome staining revealed that salidroside intervention significantly attenuated ovarian fibrosis in PCOS mice (Figure 6A). Consistent with these findings, Western blot analysis revealed the marked downregulation of α‐SMA and Collagen I in ovarian tissues (Figure 6B).

Compared with controls, PCOS mice presented elevated protein levels of LAP‐TGFβ1, TGF‐β1, and p‐SMAD2/3, all of which were effectively normalized upon salidroside treatment (Figure 6C). qRT–PCR further confirmed parallel trends in TGF‐β, α‐SMA, and Collagen I mRNA expression across the experimental groups (Figure 6D).

### 3.6 Salidroside Suppresses NLRP3‐Driven LAP‐TGFβ1 Activation to Attenuate Ovarian Fibrosis and HAS2 Dysregulation in PCOS Mice

**FIGURE 7:**
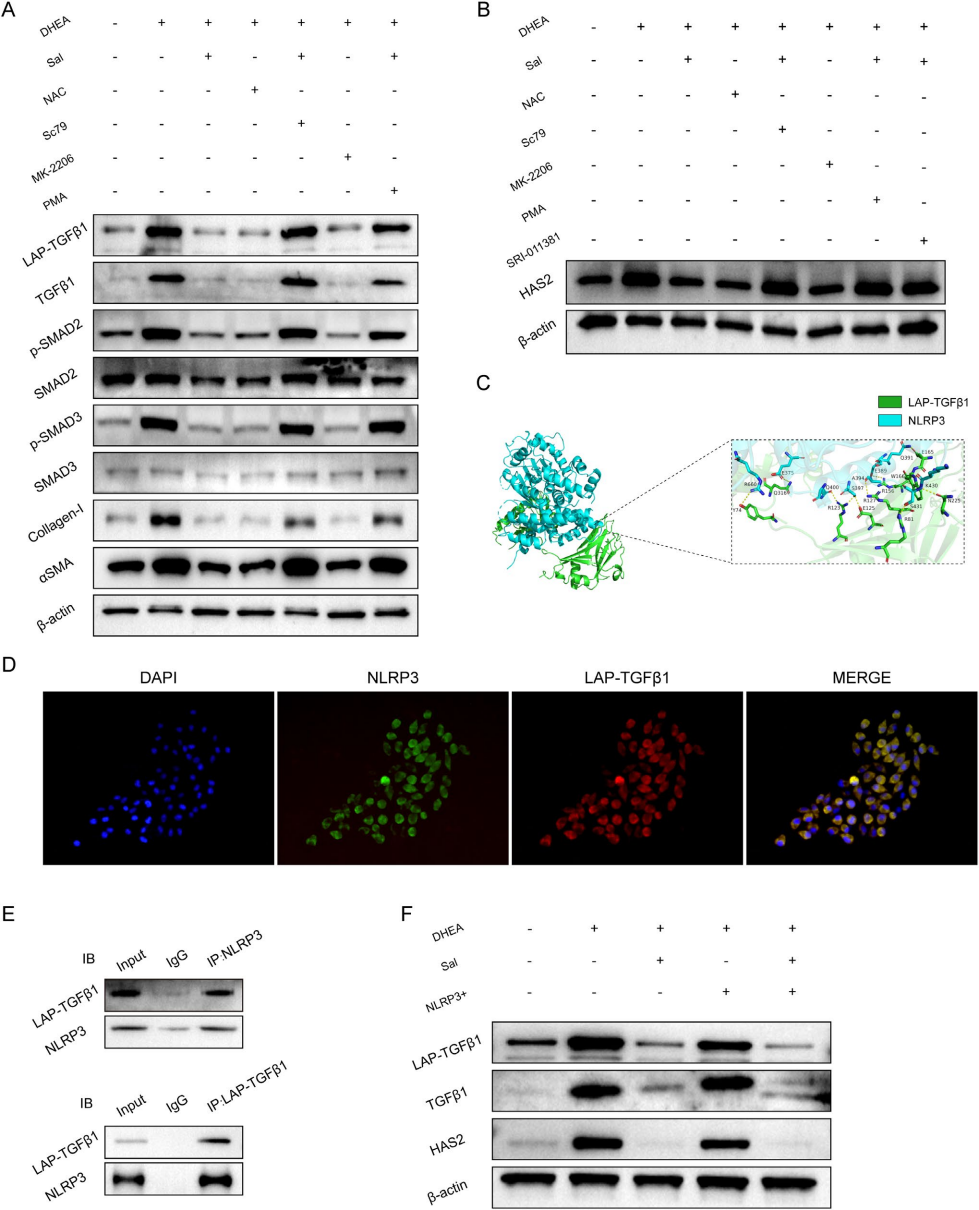
The interaction between NLRP3 and LAP‐TGF‐β1 activates the TGF‐β signaling pathway and downstream HAS2. (A) Western blot analysis of LAP‐TGF‐β1, TGF‐β1, phospho‐SMAD2, total SMAD2, phospho‐SMAD3, total SMAD3, and fibrosis‐related proteins (α‐SMA and collagen I) in GCs. (B) Western blot analysis of HAS2 protein levels in GCs. (C) Predicted molecular interaction between NLRP3 and LAP‐TGF‐β1. (D) Immunofluorescence analysis showing colocalization of LAP‐TGF‐β1 and NLRP3 in DHEA‐treated GCs. (E) Co‐IP assay to assess the interaction between LAP‐TGF‐β1 and NLRP3 in GCs. (F) Western blot analysis of LAP‐TGF‐β1, TGF‐β1, and HAS2 in NLRP3‐overexpressing GCs.

Western blot analysis of GCs revealed that the expression of proteins related to the TGF‐β‐SMAD2/3 signaling pathway changed, which was consistent with the findings in ovarian tissue (Figure 7A). Treatment with the AKT inhibitor MK‐2206 or the ROS inhibitor NAC significantly suppressed fibrosis‐related factors, whereas treatment with the AKT agonist SC79 or the NF‐κB agonist PMA abolished the therapeutic effects of salidroside. HAS2 is upregulated not only in chronic inflammation but also specifically in response to TGF‐β1 (Li et al. 2020). Treatment with the TGF‐β agonist SRI‐011381 restored HAS2 expression to levels comparable to those in the DHEA‐treated group, reversing the salidroside‐mediated alteration (Figure 7B).

TGF‐β1 activation depends on proteolytic cleavage of the latency‐associated peptide (LAP) domain in LAP‐TGFβ1 to release mature TGF‐β1 (Buscemi et al. 2011). Molecular docking predicted nine hydrogen bonding interactions between NLRP3 and LAP‐TGFβ1 (Figure 7C; docking score: −264.09; confidence score: 0.9074), indicating the potential for stable binding. To validate this interaction, dual immunofluorescence labeling confirmed the colocalization of NLRP3 and LAP‐TGF‐β1 in DHEA‐induced GCs (Figure 7D), whereas forward/reverse Co‐IP assays verified their direct interaction (Figure 7E).

Western blot analysis of NLRP3‐overexpressing GCs revealed significantly lower protein levels of HAS2, TGF‐β1, and LAP‐TGFβ1 in the Sal + DHEA group than in the DHEA group. Moreover, combined treatment with Sal + DHEA + OE‐NLRP3 reversed the OE‐NLRP3‐induced dysregulation of these molecules (Figure 7F). These data demonstrated that salidroside effectively attenuates TGF‐β‐SMAD2/3 signaling pathway activation through the suppression of the NLRP3 inflammasome and that HAS2 expression is transcriptionally modulated by interconnected inflammatory and fibrotic signaling cascades.

## 4 Discussion

Salidroside has been widely utilized in cardiovascular disease management and has well‐established pharmacological effects owing to its anti‐inflammatory and antioxidant mechanisms (Han et al. 2022). However, its therapeutic potential for treating PCOS remains insufficiently investigated. The present study mechanistically demonstrated that salidroside effectively mitigates characteristic PCOS pathologies. This is evidenced by decreased ovarian cystic follicle formation and the restoration of estrous cyclicity, whereas simultaneously normalizing the serum T level, LH level, and LH/FSH ratio.

PCOS is often accompanied by chronic low‐grade ovarian inflammation and oxidative stress (Velez et al. 2021). Patients exhibit elevated inflammatory markers in serum and follicular fluid, which promote excessive androgen production by stimulating theca cells (Jin et al. 2024; Xie et al. 2023). Consequently, anti‐inflammatory interventions are a rational therapeutic strategy. Similar to DHEA‐induced PCOS mice, lipopolysaccharide (LPS)‐stimulated chronic low‐grade inflammation models also display increased preantral and atypical follicle counts alongside reduced corpus luteum numbers (He et al. 2024; Wang et al. 2020). The present study revealed that salidroside significantly inhibits the release of the proinflammatory cytokines IL‐6, IL‐1β, and TNF‐α in PCOS models, reduces MDA levels, markedly decreases ROS levels in GCs, enhances SOD antioxidant activities, increases CAT antioxidant activities, prevents GSH depletion, and increases the GSH/GSSG ratio. The p65 subunit of NF‐κB, a key inflammatory regulator, exhibits significant phosphorylation in the ovarian GCs of PCOS patients (Liu et al. 2021). Studies have indicated that salidroside reduces the levels of phosphorylated p65 by mitigating ROS in LPS‐induced myocardial injury models (Chen et al. 2017). Consistently, the present findings revealed that salidroside effectively inhibits IκBα and p65 phosphorylation in both DHEA‐induced PCOS mice and GCs. As expected, the addition of the ROS inhibitor NAC to DHEA‐induced GCs resulted in significant reductions in the phosphorylation levels of factors in the NF‐κB pathway. These findings suggested that salidroside may prevent GC secretory dysfunction and chronic inflammation by increasing the antioxidant capacity.

Network pharmacology has been widely applied in the study of single components of drugs or traditional Chinese medicine formulations, providing critical insights into disease pathogenesis and novel strategies for personalized treatment (Nogales et al. 2022). In the present study, we utilized this approach to investigate the mechanism of salidroside in PCOS and revealed that AKT1 is the core target with the highest degree value and that the PI3K/AKT signaling pathway is a central therapeutic axis, as indicated by KEGG enrichment analysis. The PI3K/Akt pathway is a key mediator of IL‐1β‐induced inflammatory responses and apoptosis (Yang et al. 2019). Molecular docking analysis revealed a favorable binding mode of salidroside with AKT1, suggesting its potential to regulate AKT1 activity, which aligns with previous findings (Yang et al. 2023). DARTS experiments and CETSA with GCs further corroborated these results, adding to the earlier observations that salidroside significantly alleviates NF‐κB‐mediated inflammation. Furthermore, the addition of an AKT activator to GCs markedly reduces the phosphorylation levels of NF‐κB p65 and IκBα, substantiating the proposed mechanism.

ROS and phosphorylated NF‐κB act synergistically to activate the NLRP3 inflammasome, a critical driver of inflammatory responses (Zhao et al. 2024; Liu, Zhou, et al. 2025). Multiple studies have demonstrated the aberrant activation of NLRP3 in PCOS (Liu et al. 2024; Wang et al. 2020; Liu, Wang, and Wang 2025). The present findings indicated that salidroside effectively suppresses NLRP3 overactivation and that this therapeutic effect is reversible upon AKT activation. As a ubiquitous ECM component, HA mediates structural support, cellular anchorage, signal transduction regulation, and migration control in normal tissues while serving as a critical contributor to cumulus expansion (Laurent et al. 1996; Jiang et al. 2007, 2011). In the present study, we focused on HAS2, the key enzyme governing HA biosynthesis during animal reproduction, which directly determines HA abundance (Zhang et al. 2023). Recent studies have paradoxically demonstrated that elevated HA levels in the ovaries of PCOS mice correlate with exacerbated inflammation, which contrasts with the well‐documented physiological benefits of HA (He et al. 2024). Emerging evidence confirms the involvement of HA in inflammatory responses and immunomodulatory processes (Homann et al. 2018). The present study further revealed aberrant HAS2 upregulation in both PCOS mouse ovaries and DHEA‐treated GCs, which was effectively reversed by salidroside intervention. Mechanistic investigations in GCs revealed that AKT/NF‐κB signaling is a regulatory pathway that controls HAS2 expression.

Chronic inflammation‐associated fibrosis has long been a challenging issue in the ovaries of PCOS patients (Xue et al. 2022). NLRP3 promotes ovarian fibrosis by activating pyroptosis and the TGFβ‐SMAD2/3 signaling pathway, thereby inducing the activation of ECM‐associated proteins closely linked to fibrosis including TGFβ1, α‐SMA, and Collagen I (Wang et al. 2020). The present study demonstrated that salidroside treatment effectively reverses these pathological changes. Mechanistically, we confirmed that TGFβ activation and expression are regulated by NLRP3. TGF‐β1 activation requires cleavage of the latency‐associated peptide (LAP) domain of its precursor protein, a process mediated through plasmin‐mediated proteolysis, integrin αV binding, oxidative modification, or thrombospondin‐1 interactions (Shi et al. 2011). Activated TGF‐β1 binds to the TGF‐βRII/TGF‐βRI receptor complex, triggering Smad2/3 phosphorylation and nuclear translocation, which subsequently leads to the formation of a transcriptional complex with Smad4 to regulate downstream target genes (Chia et al. 2024; Felipe et al. 2019). This transcriptional activation ultimately drives alterations in TGF‐β‐regulated cellular functions and gene expression. Therefore, the abnormal activation and overexpression of TGF‐β1 constitute critical mechanisms underlying fibrosis initiation and progression.

The present study first revealed that NLRP3 directly interacts with LAP‐TGFβ1 in DHEA‐induced GCs to activate TGFβ1. The production of HA, a major ECM component, is also regulated by the TGFβ‐SMAD2/3 signaling pathway (Wang et al. 2019). Both TGFβ activator treatment and NLRP3 overexpression significantly increase HAS2 expression.

**FIGURE 8:**
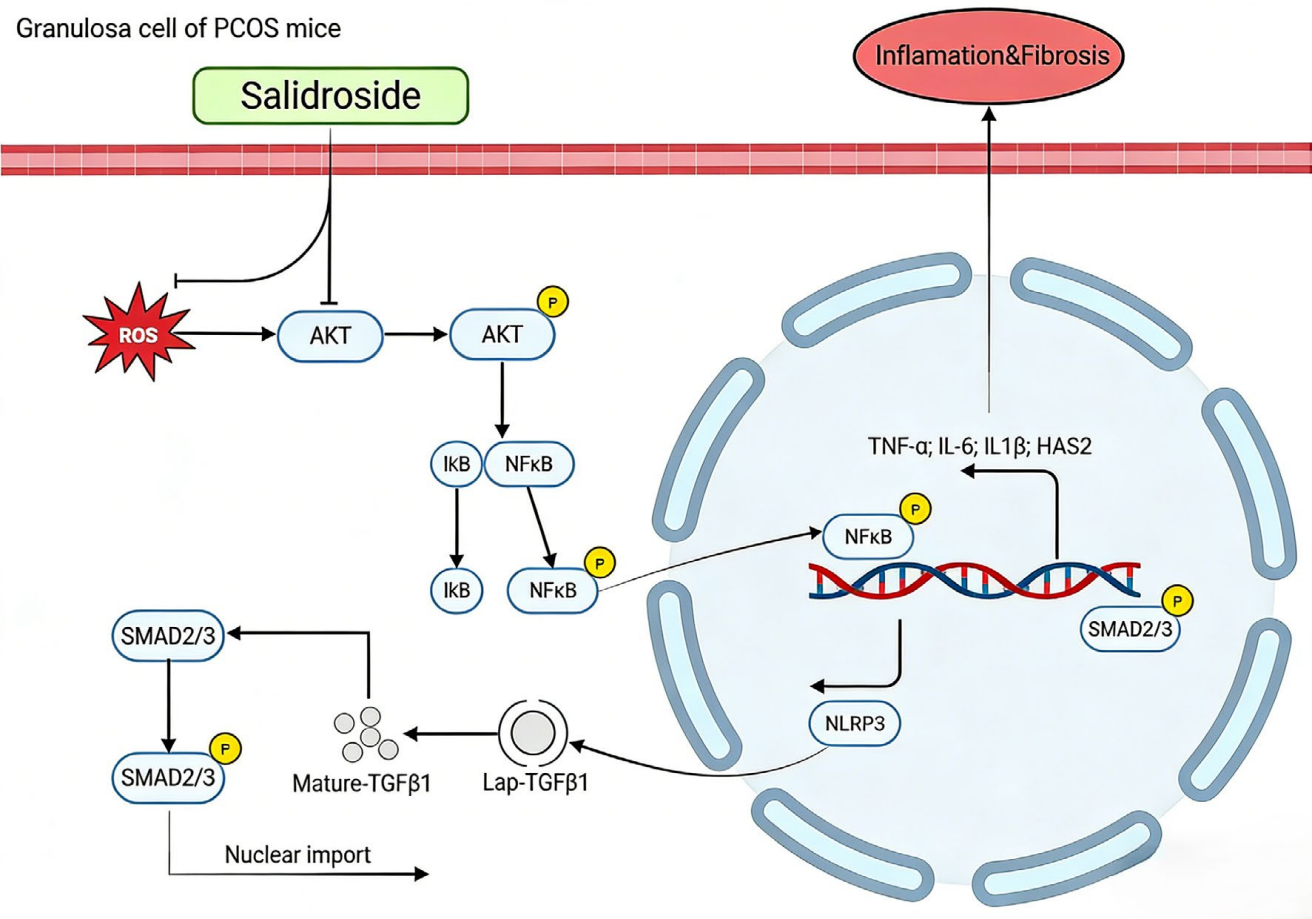
The mechanisms of salidroside in the treatment of PCOS. Salidroside ameliorates PCOS via coordinated AKT/NF‐κB/NLRP3 signaling suppression and HAS2 downregulation.

HA typically exists as high‐molecular‐weight polymers (> 1000 kDa) under physiological conditions, but it can be degraded into low‐molecular‐weight forms under pathological conditions (e.g., tissue injury or inflammation) (Rowley et al. 2020). Emerging evidence suggests that the biological properties of HA depend on its molecular weight, potentially explaining the contradictory characteristics observed in different contexts (Małgorzata et al. 2016; Saari and Konttinen 1989). Further investigations are needed to clarify HA degradation patterns in PCOS tissues, which may represent another therapeutic target. Taken together, the present results established salidroside as a promising therapeutic candidate for PCOS and elucidated its unique mechanism, which involves modulation the AKT/NF‐κB/NLRP3 signaling axis and TGFβ‐SMAD2/3 pathway‐mediated HA overproduction (Figure 8).

This study may have several limitations. First, the identification of salidroside's core target AKT relied on network pharmacology. Inherent differences in the algorithms and coverage of the various databases used may have led to the omission of other potential targets, potentially introducing bias into the target selection process. Second, although CETSA and DARTS assays suggested a direct interaction, the critical validation using AKT point mutation models (e.g., K179M) to confirm binding specificity and necessity was not performed. This gap limits the conclusive evidence for salidroside acting as a direct and specific AKT inhibitor. Third, while salidroside effectively ameliorated ovarian pathology and inflammation, its impact on the broader systemic metabolic dysfunctions (such as insulin resistance and glucose homeostasis) commonly associated with PCOS was not thoroughly investigated. Future studies integrating these aspects are needed to fully elucidate salidroside's potential as a comprehensive therapeutic agent for PCOS.

## 5 Conclusion

In conclusion, our findings suggested that salidroside may alleviate DHEA‐induced PCOS symptoms in mice, potentially by targeting the AKT/NF‐κB/NLRP3 signaling axis to suppress ovarian oxidative stress, inflammation, and fibrosis. The downregulation of HAS2 emerged as a potential link connecting these inflammatory signals to aberrant extracellular matrix remodeling in the PCOS ovary. This study provides preclinical evidence highlighting salidroside as a natural compound worthy of further investigation for PCOS, whereas also underscoring the need to validate its precise molecular targets and therapeutic efficacy in more comprehensive models.

## Author Contributions

**Jing‐hang Li:** conceptualization. **Zhi‐Chao Chi:** data curation. **Dong‐lei Yan:** software. **Jia‐hui Leng:** validation. **Hong‐jie Xu:** visualization. **Lin‐Yi Qu:** resources. **Guan‐Lin Jia:** writing – original draft. **Li‐Ying Liu:** methodology. **Tian Lan:** supervision. **Yu‐Yan He:** investigation. **Xian‐Feng Yu:** project administration. **Il‐keun Kong:** writing – review and editing. **Ming‐Jun Zhang:** formal analysis. **Yong‐Xun Jin:** funding acquisition. **Shu‐Ming Shi:** formal analysis.

## Funding

This research was funded by the earmarked fund for Jilin Provincial Natural Science Foundation of China (20250205022GH).

## Ethics Statement

All experiments on animals were performed under the Ethical Welfare Committee of Jilin University's endorsement (no. SY202409305).

## Conflicts of Interest

The authors declare no conflicts of interest.

## Supporting information


**Table S1:** Primary antibodies of Western blot.
**Table S2:** Sequences of oligonucleotide primers for qRT‐PCR.
**Figure S1:** Effects of salidroside on oxidative stress in DHEA or salidroside treated GCs.

## Data Availability

The data that support the findings of this study are available from the corresponding author upon reasonable request.
