# Supplementary material for: Salidroside Ameliorates Polycystic Ovary Syndrome in Mice by Regulating the AKT/NF‐κB/NLRP3‐HAS2 Axis
Source: Food Sci Nutr. 2026 Apr 4;14(4):e71692. doi: 10.1002/fsn3.71692 (PMC13052134; doi:10.1002/fsn3.71692)
Supplement: Supplementary file 1 — Table S1: Primary antibodies of Western blot. Table S2: Sequences of oligonucleotide primers for qRT‐PCR. Figure S1: Effects of salidroside on oxidative stress in DHEA or salidroside treated GCs. [file FSN3-14-e71692-s001.docx]

**Supplemental Table**

**Supplementary Table S1** Primary antibodies of Western blot

| Primary antibody | Catalog No. | Dilution | Co.,Ltd |
| --- | --- | --- | --- |
| p-AKT | 4060 | 1:2000 | Cell Signaling Technology |
| AKT | 9272 | 1:2000 | Cell Signaling Technology |
| p-P65 | 3033 | 1:1000 | Cell Signaling Technology |
| P65 | 8242 | 1:2000 | Cell Signaling Technology |
| p-IκBα | [TA2002](http://www.ab-mart.com.cn/page.aspx?node=%2077%20&id=%2018305) | 1:1000 | Abmart |
| IκBα | [T55026](http://www.ab-mart.com.cn/page.aspx?node=%2077%20&id=%201330) | 1:1000 | Abmart |
| NLRP3 | CY5651 | 1:1000 | Abways |
| HAS2 | PA5-115388 | 1:2000 | ThermoFisher |
| TGFβ1 | ab179695 | 1:2000 | Abcam |
| p-SMAD2 | CY5857 | 1:2000 | Abways |
| SMAD2 | T55090 | 1:2000 | Abmart |
| p-SMAD3 | 9520 | 1:2000 | Cell Signaling Technology |
| SMAD3 | 9513 | 1:2000 | Cell Signaling Technology |
| α-SMA | T55295 | 1:2000 | Abmart |
| Collagen I | CY5120 | 1:1000 | Abways |
| β-actin | ABL1010 | 1:2000 | Abkkine |

**Supplementary Table S2** Sequences of oligonucleotide primers for qRT-PCR.

| Gene name | Forward (5'→3') | Reverse (5'→3') |
| --- | --- | --- |
| ACTB | GGCTGTATTCCCCTCCATCG | CCAGTTGGTAACAATGCCATGT |
| TGF-β | CTCCCGTGGCTTCTAGTGC | GCCTTAGTTTGGACAGGATCTG |
| αSMA | GATGCTCCCCGGGCTCTATG | CATGATCTGGGTCATCTTCTCAC |
| COL1A1 | GCTCCTCTTAGGGGCCACT | CCACGTCTCACCATTGGGG |
| IL-6 | TAGTCCTTCCTACCCCAATTTCC | TTGGTCCTTAGCCACTCCTTC |
| IL-1β | GCAACTGTTCCTGAACTCAACT | ATCTTTTGGGGTCCGTCAACT |
| TNF-α | CATCTTCTCAAAATTCGAGTGACAA | TGGGAGTAGACAAGGTACAACCC |

**Supplemental Figure**

**
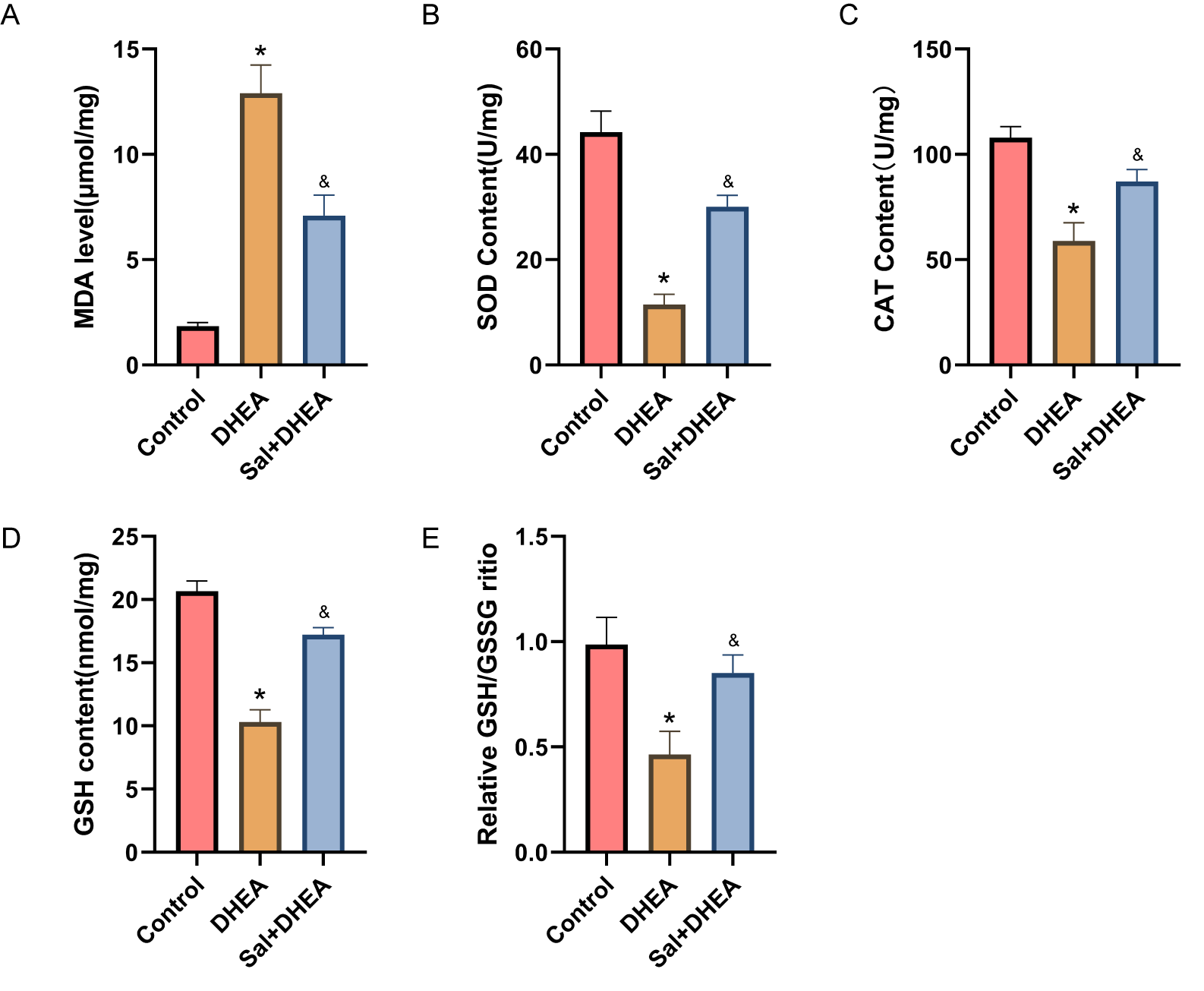
**

**Supplementary Table S1** Effects of salidroside on oxidative stress in DHEA or Salidroside treated GCs. (A) GCs MDA contents. (B, C) GCs SOD and CAT activities. (D, E) GCs GSH content and GSH/GSSG ratio.
